# Supplementary material for: Uncovering diversity and metabolic spectrum of animals in dead zone sediments
Source: Commun Biol. 2020 Mar 6;3:106. doi: 10.1038/s42003-020-0822-7 (PMC7060179; doi:10.1038/s42003-020-0822-7)
Supplement: Supplementary file 1 — Supplementary Information [file 42003_2020_822_MOESM1_ESM.docx]

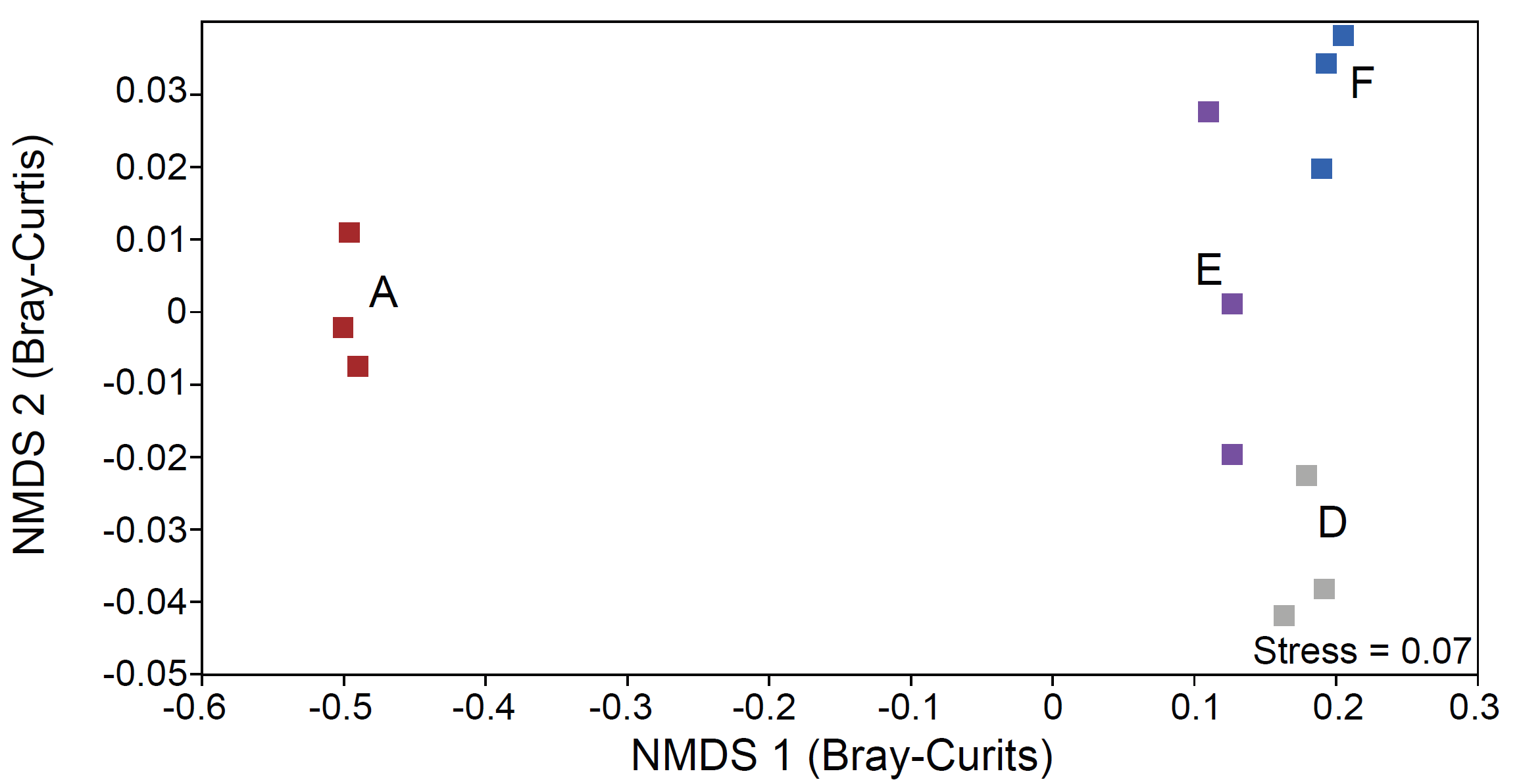


**Supplementary Figure 1** NMDS of the Bray-Curtis dissimilarity based on the relative abundance of the SILVA classified 18S rRNA eukaryotic community composition for RNA samples. The colors denote sediment samples from stations A (brown), D (grey), E (purple), and F (blue).

**
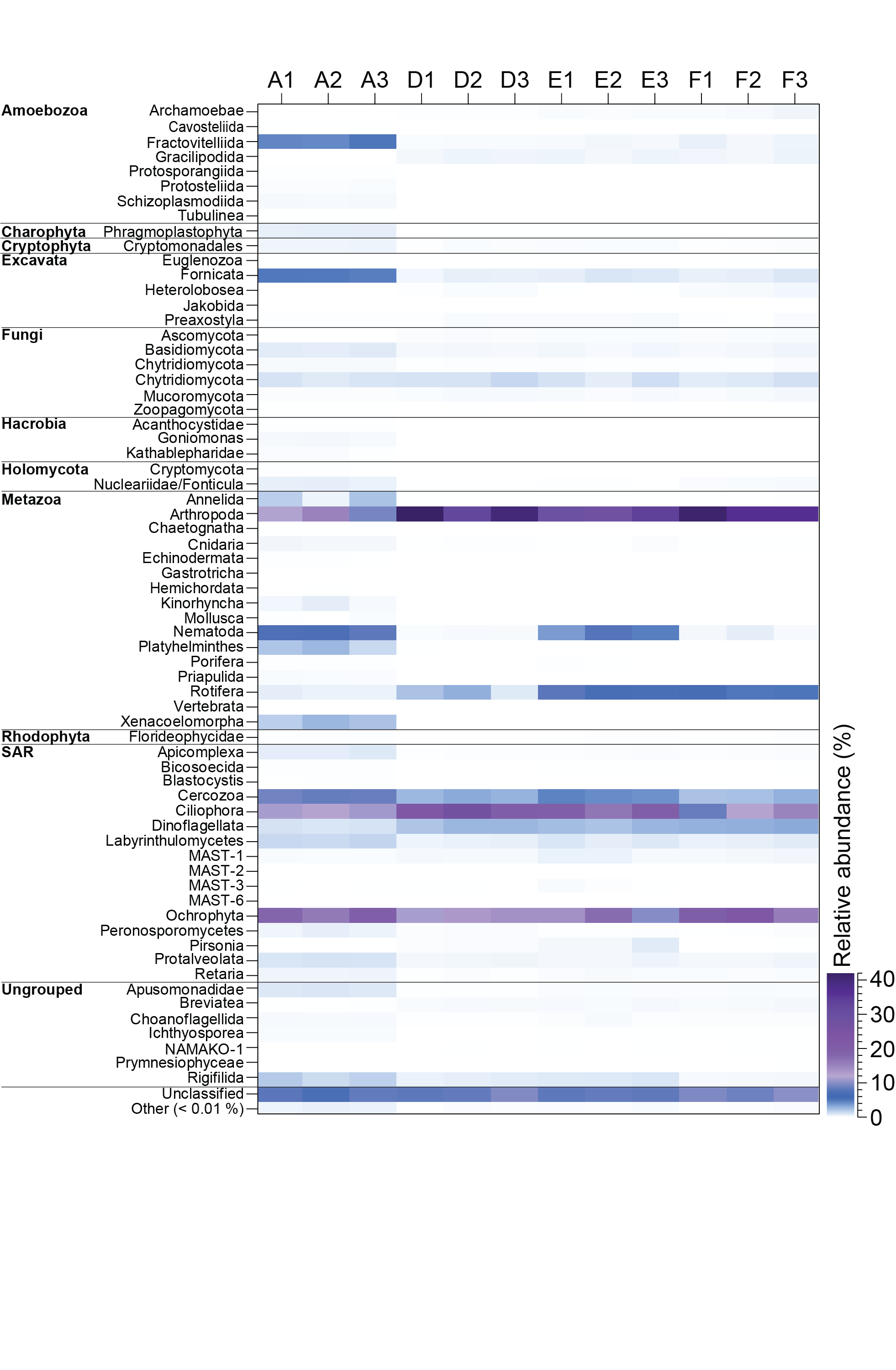
**

**Supplementary Figure 2** Eukaryotic 18S rRNA community composition based on extracted 18S rRNA sequences from the total RNA-seq (based on results from SILVA taxonomic classifications). The heatmap shows taxonomy groups > 0.01 % (average of all samples; groups < 0.01 % are denoted as “Other” in the figure). Taxa were grouped into Amoebozoa, Charophyta Cryptophyta, Excavata, Fungi, Hacrobia, Holomycota, Metazoa, Rhodophyta, and SAR. The colors denote relative abundances with white representing 0 %, white-blue gradient 0-6 %, blue-purple gradient medium 6-12 %, and light purple-dark purple gradient 12-42 %.


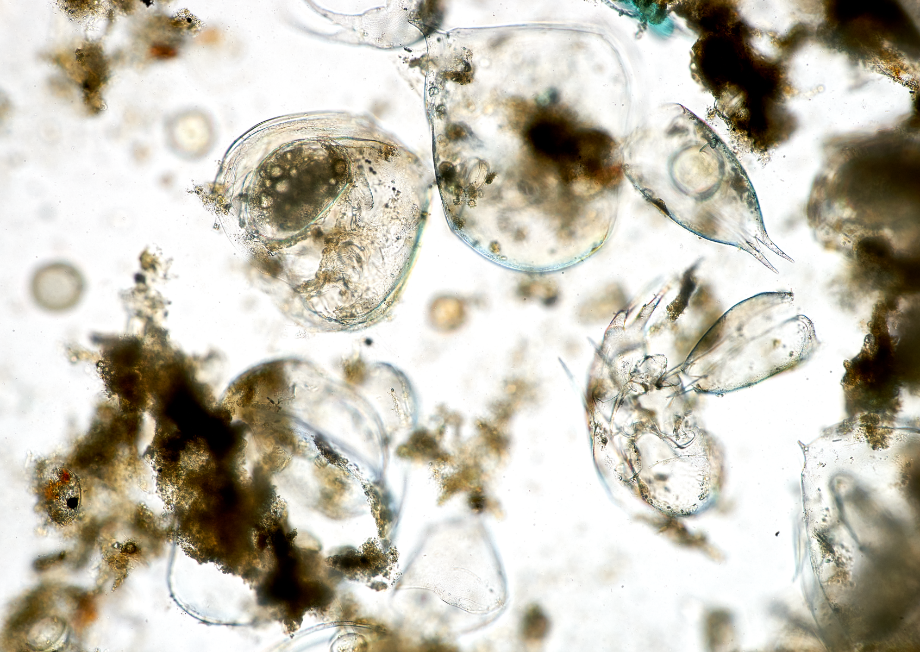

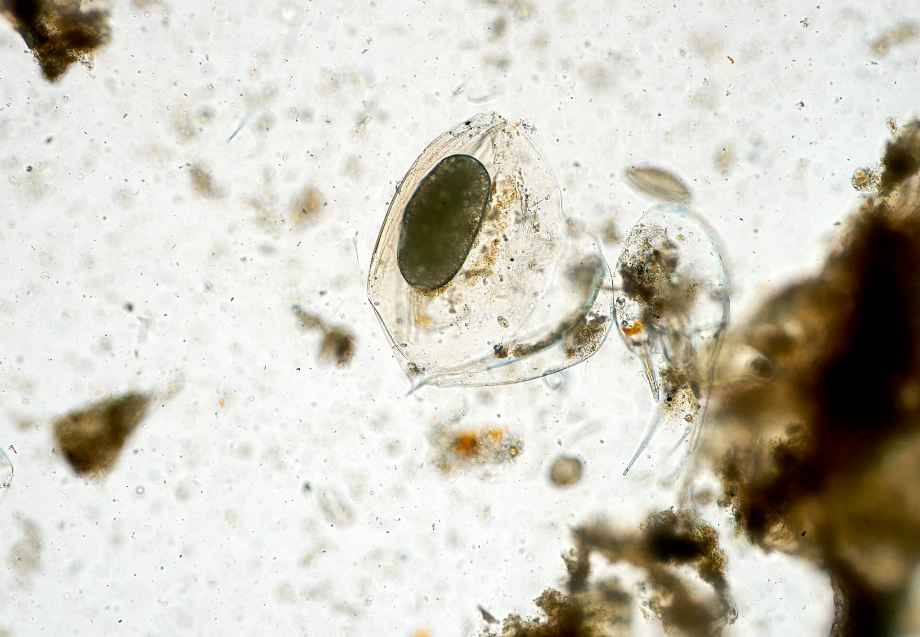


**Supplementary Figure 3** Microscopy images of Bosminidae-like zooplankton detected in the sediment at Station E.


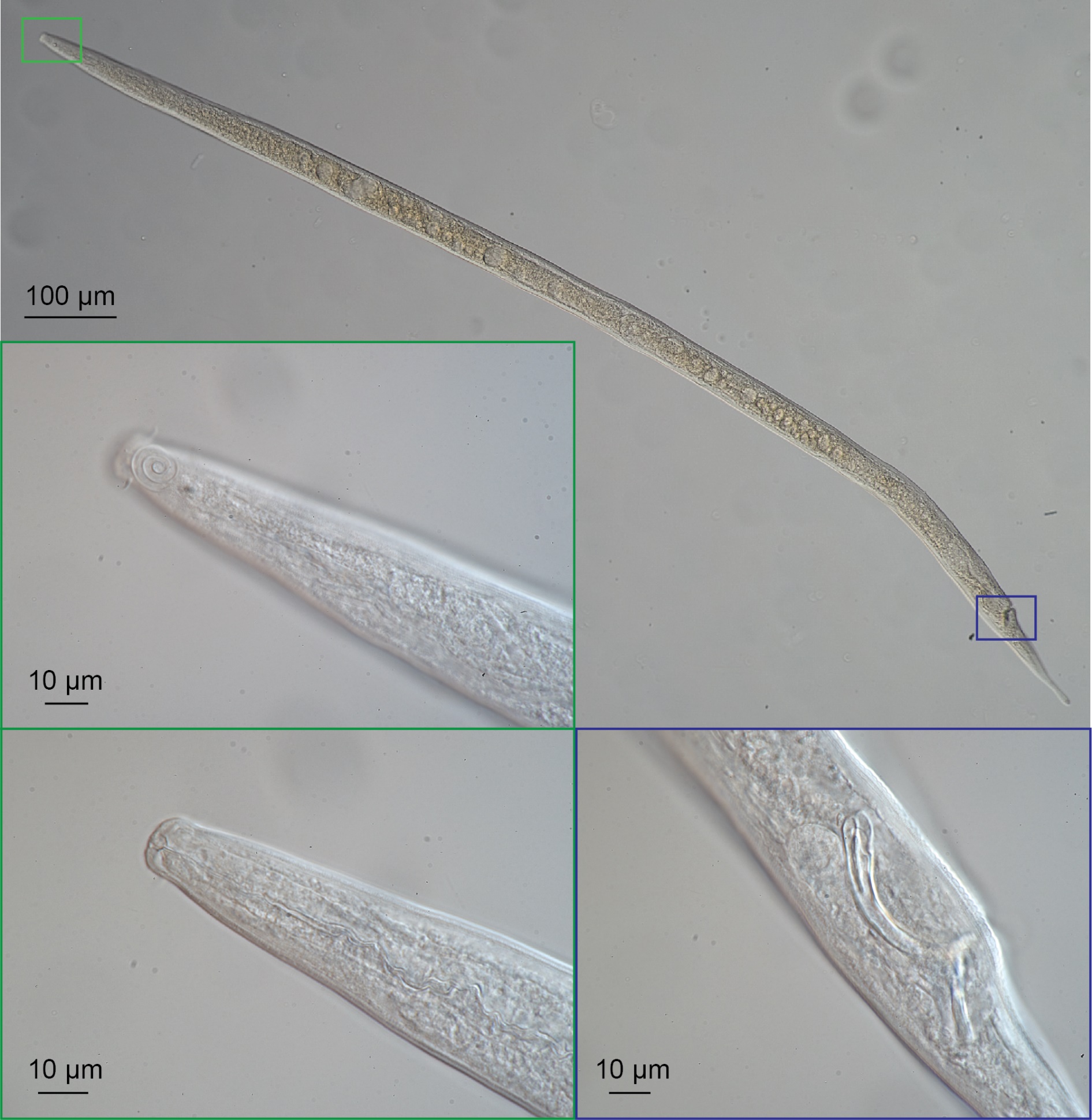


**Supplementary Figure 4** Microscopy image of one adult male *Sabatieria* collected from sediment at station E. The green boxes show details of the amphid (top) and buccal cavity (bottom), while the blue box shows detail of the copulatory spicules.

**Supplementary Table 1** Spearman correlations based on the measured O_2_, H_2_O, and N_2_O microprofiles in the sediment cores. The input data was based on measurements from similar µm depths from all replicate cores across all stations. Yellow cells show statistically significant results.

|  |  | O2 | H2S | N2O |
| --- | --- | --- | --- | --- |
| O2 | rho | 1 | -.783^**^ | .437^**^ |
|  | *P* |  | 0.0000000000000000000000029 | 0.00000054 |
|  | *n* | 221 | 111 | 121 |
| H2S | rho | -.783** | 1 | -.782^**^ |
|  | *P* | 0.0000000000000000000000029 |  | 0.00000000000000000042 |
|  | *n* | 111 | 176 | 87 |
| N2O | rho | .437** | -.782^**^ | 1 |
|  | *P* | 0.00000054 | 0.00000000000000000042 |  |
|  | *n* | 121 | 87 | 121 |

** Correlation is significant at the 0.01 level (2-tailed).

**Supplementary Table 2** Shannon’s H alpha diversity index for all samples in all datasets (18S rRNA SILVA and 18S rRNA NCBI NT). Before analysis the data was bootstrapped × 100 to the minimum sample size 3,486,047, and 4,312,510 counts respectively. Minimum sample size for the Nematoda 18S rRNA NCBI NT data was 12,776 counts. The table shows the bootstrap mean of Shannon’s H.

| **RNA samples** | **18S rRNA SILVA** | **18S rRNA NCBI NT** | | **Nematodes 18S rRNA NCBI NT** | |
| --- | --- | --- | --- | --- | --- |
| RNA A1 | 7.58 | 5.09 | | 4.57 | |
| RNA A2 | 7.50 | 5.38 | | 4.57 | |
| RNA A3 | 7.47 | 4.86 | | 4.03 | |
| RNA D1 | 4.80 | 2.66 | | 4.45 | |
| RNA D2 | 5.29 | 2.62 | | 4.25 | |
| RNA D3 | 5.01 | 2.60 | | 3.91 | |
| RNA E1 | 5.55 | 2.65 | | 2.00 | |
| RNA E2 | 5.63 | 2.60 | | 1.53 | |
| RNA E3 | 5.61 | 3.03 | | 2.55 | |
| RNA F1 | 4.64 | 2.48 | | 3.14 | |
| RNA F2 | 4.80 | 2.38 | | 3.38 | |
| RNA F3 | 5.10 | 2.63 | | 4.18 | |
|  |  |  |  | |  |
| **Sub-sample size** | Counts |  |  | |  |
| 18S rRNA SILVA | 3,486,047 |  |  | |  |
| 18S rRNA NCBI NT | 4,312,510 |  |  | |  |
| Nematodes 18S rRNA NCBI NT | 12,776 |  |  | |  |

**Supplementary Table 3** Bosminidae RNA transcripts successfully annotated against the NCBI NR database and accession numbers linked to the InterPro protein database. Results for the family Bosminidae (including genera *Bosmina* and *Eubosmina*) were extracted from the software MEGAN and is shown in the table. Values shown in the table are thousands of CPM for the family Bosminidae (i.e. CPM × 10^-3^), and the color gradient in grey shows the lowest number as white (0) and the highest number as dark grey (1000). Dashes denote no data.

|  | **InterPro ID** | **Protein family** | A1 | A2 | A3 | D1 | D2 | D3 | E1 | E2 | E3 | F1 | F2 | F3 |
| --- | --- | --- | --- | --- | --- | --- | --- | --- | --- | --- | --- | --- | --- | --- |
| Bosminidae | IPR000883 | Cytochrome c oxidase subunit I | - | 444 | 0 | 563 | 800 | 520 | 310 | 545 | 452 | 338 | - | 606 |
|  | IPR003917 | NADH:ubiquinone oxidoreductase, chain 2 | - | 444 | 1000 | 250 | 133 | 360 | 571 | 455 | 405 | 477 | - | 212 |
|  | IPR001404 | Heat shock protein Hsp90 family | - | 0 | 0 | 94 | 0 | 100 | 71 | 0 | 48 | 169 | - | 121 |
|  | IPR022471 | Cysteine desulphurase, catalytic subunit, CsdA | - | 111 | 0 | 94 | 67 | 20 | 48 | 0 | 95 | 15 | - | 61 |

**Supplementary Table 4** Rotifera RNA transcripts successfully annotated against the NCBI NR database and accession numbers linked to the InterPro protein database. Results for Rotifera were extracted from the software MEGAN and is shown in the table. Data was delimited to CPM values > 50 000 in at least one sample. Values shown in the table are thousands of CPM for the Rotifera (i.e. CPM × 10^-3^), and the color gradient in grey shows the lowest number as white (0) and the highest number as dark grey (208).

|  | **InterPro ID** | **Protein family** | A1 | A2 | A3 | D1 | D2 | D3 | E1 | E2 | E3 | F1 | F2 | F3 |
| --- | --- | --- | --- | --- | --- | --- | --- | --- | --- | --- | --- | --- | --- | --- |
| Rotifera (> 50 000 CPM in at least one sample) | IPR031107 | Small heat shock protein HSP20 | 63 | 0 | 67 | 140 | 140 | 42 | 166 | 101 | 115 | 121 | 108 | 114 |
|  | IPR000883 | Cytochrome c oxidase subunit I | 63 | 0 | 67 | 0 | 96 | 0 | 36 | 74 | 50 | 64 | 54 | 38 |
|  | IPR030689 | Cytochrome b | 42 | 54 | 0 | 65 | 44 | 42 | 23 | 32 | 40 | 27 | 8 | 42 |
|  | IPR001947 | Scorpion short chain toxin, potassium channel inhibitor | 0 | 0 | 0 | 47 | 74 | 208 | 8 | 5 | 40 | 11 | 8 | 17 |
|  | IPR024791 | Cytochrome c oxidase subunit III | 0 | 0 | 0 | 9 | 29 | 83 | 10 | 0 | 15 | 27 | 15 | 0 |
|  | IPR000568 | ATP synthase, F0 complex, subunit A | 0 | 0 | 0 | 37 | 37 | 0 | 8 | 0 | 25 | 15 | 54 | 13 |
|  | IPR028460 | Tyramine beta-hydroxylase/Dopamine beta-hydroxylase | 0 | 0 | 0 | 0 | 0 | 125 | 0 | 0 | 0 | 0 | 0 | 0 |
|  | IPR031327 | Mini-chromosome maintenance protein | 0 | 0 | 67 | 0 | 0 | 0 | 3 | 11 | 30 | 0 | 0 | 4 |
|  | IPR000990 | Innexin | 42 | 0 | 0 | 56 | 7 | 0 | 5 | 0 | 0 | 0 | 0 | 0 |
|  | IPR013078 | Histidine phosphatase superfamily, clade-1 | 104 | 0 | 0 | 0 | 0 | 0 | 0 | 0 | 0 | 0 | 0 | 0 |
|  | IPR000322 | Glycoside hydrolase family 31 | 0 | 0 | 0 | 0 | 0 | 83 | 0 | 0 | 0 | 0 | 0 | 17 |
|  | IPR000850 | Adenylate kinase/UMP-CMP kinase | 0 | 0 | 100 | 0 | 0 | 0 | 0 | 0 | 0 | 0 | 0 | 0 |
|  | IPR002455 | GPCR family 3, GABA-B receptor | 0 | 0 | 100 | 0 | 0 | 0 | 0 | 0 | 0 | 0 | 0 | 0 |
|  | IPR015925 | Ryanodine receptor-related | 0 | 0 | 100 | 0 | 0 | 0 | 0 | 0 | 0 | 0 | 0 | 0 |
|  | IPR000751 | M-phase inducer phosphatase | 0 | 81 | 0 | 0 | 0 | 0 | 3 | 0 | 0 | 0 | 0 | 0 |
|  | IPR033756 | Flagellum site-determining protein YlxH | 0 | 0 | 0 | 0 | 0 | 83 | 0 | 0 | 0 | 0 | 0 | 0 |
|  | IPR009621 | Uncharacterised protein family UPF0239 | 0 | 0 | 0 | 0 | 0 | 83 | 0 | 0 | 0 | 0 | 0 | 0 |
|  | IPR000720 | Peptidylglycine alpha-hydroxylating monooxygenase | 63 | 0 | 0 | 0 | 0 | 0 | 0 | 5 | 5 | 0 | 0 | 0 |
|  | IPR024990 | Anaphase-promoting complex subunit 1 | 0 | 0 | 67 | 0 | 0 | 0 | 0 | 0 | 5 | 0 | 0 | 0 |
|  | IPR006035 | Ureohydrolase | 0 | 0 | 67 | 0 | 0 | 0 | 0 | 0 | 5 | 0 | 0 | 0 |
|  | IPR000276 | G protein-coupled receptor, rhodopsin-like | 0 | 0 | 67 | 0 | 0 | 0 | 0 | 0 | 0 | 0 | 0 | 4 |
|  | IPR010286 | Ribosomal RNA large subunit methyltransferase F-like | 0 | 0 | 67 | 0 | 0 | 0 | 0 | 0 | 0 | 0 | 0 | 0 |
|  | IPR026306 | RSBN1/Dpy-21 | 0 | 54 | 0 | 0 | 0 | 0 | 0 | 0 | 0 | 0 | 8 | 4 |
|  | IPR016491 | Septin | 63 | 0 | 0 | 0 | 0 | 0 | 0 | 0 | 0 | 0 | 0 | 0 |
|  | IPR016901 | Anaphase-promoting complex subunit APC10/Doc1 | 0 | 0 | 0 | 0 | 0 | 0 | 0 | 0 | 0 | 0 | 62 | 0 |
|  | IPR001392 | Clathrin adaptor, mu subunit | 0 | 54 | 0 | 0 | 0 | 0 | 0 | 0 | 0 | 0 | 0 | 0 |
|  | IPR013956 | E3 ubiquitin ligase Bre1 | 0 | 54 | 0 | 0 | 0 | 0 | 0 | 0 | 0 | 0 | 0 | 0 |
|  | IPR000924 | Glutamyl/glutaminyl-tRNA synthetase | 0 | 54 | 0 | 0 | 0 | 0 | 0 | 0 | 0 | 0 | 0 | 0 |
|  | IPR010122 | Hydroxymethylglutaryl-CoA synthase, eukaryotic | 0 | 54 | 0 | 0 | 0 | 0 | 0 | 0 | 0 | 0 | 0 | 0 |
|  | IPR024768 | Meiosis regulator and mRNA stability factor 1 | 0 | 54 | 0 | 0 | 0 | 0 | 0 | 0 | 0 | 0 | 0 | 0 |
|  | IPR007271 | Nucleotide-sugar transporter | 0 | 54 | 0 | 0 | 0 | 0 | 0 | 0 | 0 | 0 | 0 | 0 |
|  | IPR034911 | Polyadenylate-binding protein 2 | 0 | 54 | 0 | 0 | 0 | 0 | 0 | 0 | 0 | 0 | 0 | 0 |
|  | IPR027684 | Tubulin-specific chaperone C | 0 | 54 | 0 | 0 | 0 | 0 | 0 | 0 | 0 | 0 | 0 | 0 |
|  |  | Total amount of proteins classified | 26 | 25 | 17 | 35 | 51 | 14 | 134 | 94 | 90 | 88 | 58 | 99 |
