# Supplementary material for: Uncovering diversity and metabolic spectrum of animals in dead zone sediments
Source: Commun Biol. 2020 Mar 6;3:106. doi: 10.1038/s42003-020-0822-7 (PMC7060179; doi:10.1038/s42003-020-0822-7)
Supplement: Supplementary file 2 — Description of Additional Supplementary Items [file 42003_2020_822_MOESM2_ESM.docx]

**Description of Additional Supplementary Items**

**Supplementary Data 1** O_2_, N_2_O, and H_2_S concentrations in the different sediment depths measured by microprofiling and used in the correlation analysis.

**Supplementary Data 2** Taxonomic classification of eukaryotic 18S rRNA sequencing against the SILVA database. The Data shows raw counts.

**Supplementary Data 3** Taxonomic classification of eukaryotic 18S rRNA sequencing against the NCBI NT database. The Data shows raw counts.

**Supplementary Data 4** The excel file shows the Eukaryotic results from the annotation of RNA transcripts (i.e. SortMeRNA extracted non-rRNA sequences) against the NCBI NR database using the aligner Diamond. Accession numbers linked to taxonomy are shown in sheet 1, while sheet 2, 3, and 4 shows proteins affiliated with the family Bosminidae, phyla Nematoda, and phyla Rotifera, respectively. Values shown in the tables are counts per million sequences (CPM) for all eukaryota (sheet 1), and for each taxonomic group in sheet 2, 3, and 4. In addition, the average of CPM for all samples, and the raw counts are shown in each sheet.

**Supplementary Data 5** Information from the sequencing round with the sequencing family IDs, amount of sequences before and after quality trimming, average read length, and quality scores. In addition, information from the bioinformatic analyses is provided with amount of rRNA sequences, and the proportion of sequences taxonomically and metabolically classified.

**Supplementary Data 6** The excel file shows all details from the ANOVA tests and post hoc Tukey tests.
