# Supplementary material for: Uncovering diversity and metabolic spectrum of animals in dead zone sediments
Source: Commun Biol. 2020 Mar 6;3:106. doi: 10.1038/s42003-020-0822-7 (PMC7060179; doi:10.1038/s42003-020-0822-7)
Supplement: Supplementary file 3 — Reporting Summary [file 42003_2020_822_MOESM3_ESM.pdf]

nature research

Corresponding author(s): Elias Broman

Last updated by author(s): Jan 29, 2020

Reporting Summary

Nature Research wishes to improve the reproducibility of the work that we publish. This form provides structure for consistency and transparency in reporting. For further information on Nature Research policies, see Authors & Referees and the Editorial Policy Checklist.

Statistics

For all statistical analyses, confirm that the following items are present in the figure legend, table legend, main text, or Methods section.

n/a	Confirmed	
☐	☒	The exact sample size (*n*) for each experimental group/condition, given as a discrete number and unit of measurement
☐	☒	A statement on whether measurements were taken from distinct samples or whether the same sample was measured repeatedly
☐	☒	The statistical test(s) used AND whether they are one- or two-sided<br>*Only common tests should be described solely by name; describe more complex techniques in the Methods section.*
☒	☐	A description of all covariates tested
☐	☒	A description of any assumptions or corrections, such as tests of normality and adjustment for multiple comparisons
☐	☒	A full description of the statistical parameters including central tendency (e.g. means) or other basic estimates (e.g. regression coefficient) AND variation (e.g. standard deviation) or associated estimates of uncertainty (e.g. confidence intervals)
☐	☒	For null hypothesis testing, the test statistic (e.g. *F*, *t*, *r*) with confidence intervals, effect sizes, degrees of freedom and *P* value noted<br>*Give P values as exact values whenever suitable.*
☒	☐	For Bayesian analysis, information on the choice of priors and Markov chain Monte Carlo settings
☒	☐	For hierarchical and complex designs, identification of the appropriate level for tests and full reporting of outcomes
☐	☒	Estimates of effect sizes (e.g. Cohen's *d*, Pearson's *r*), indicating how they were calculated

Our web collection on statistics for biologists contains articles on many of the points above.

Software and code

Policy information about availability of computer code

Data collection	Data was collected from field samples.
Data analysis	Plotting of the profile graphs were carried out using SigmaPlot v. 14 (Systat Software, USA). Statistics of molecular data was conducted in R, SPSS, past, and Explicet.

For manuscripts utilizing custom algorithms or software that are central to the research but not yet described in published literature, software must be made available to editors/reviewers. We strongly encourage code deposition in a community repository (e.g. GitHub). See the Nature Research guidelines for submitting code & software for further information.

Data

Policy information about availability of data

All manuscripts must include a data availability statement. This statement should provide the following information, where applicable:

- Accession codes, unique identifiers, or web links for publicly available datasets
- A list of figures that have associated raw data
- A description of any restrictions on data availability

The data that support these findings are available in the manuscript and supplemental files. The raw sequence data has been deposited online and can be accessed at the NCBI BioProject PRJNA531756.

Field-specific reporting

Please select the one below that is the best fit for your research. If you are not sure, read the appropriate sections before making your selection.

☐ Life sciences ☐ Behavioural & social sciences ☒ Ecological, evolutionary & environmental sciences

For a reference copy of the document with all sections, see nature.com/documents/nr-reporting-summary-flat.pdf

Ecological, evolutionary & environmental sciences study design

All studies must disclose on these points even when the disclosure is negative.

Study description	This study aimed to use the most advanced geochemical and molecular methods to increase our understanding of micrometazoan diversity and metabolism in low oxygen and sulfidic environments.
Research sample	Sediment was sampled from the Eastern Gotland Basin located in the Baltic Sea.
Sampling strategy	Based on previous results from the same sampling stations showing extremely low spatial variability in biogeochemical and microbiological properties of this dead zone sediment (Marzocchi et al 2018. Environmental microbiology), we collected triplicate cores, which assured enough replication.
Data collection	A research cruise was conducted in April 2018. Water and sediment samples were collected at four stations along a depth gradient in the Baltic Sea (see manuscript Fig. 1). Oxygen depth profiles in the water column were obtained by CTD casting (SBE911, USA) while water samples were collected with a Rosette sampler carrying 10 l Niskin bottles. Sediment was sampled via a boxcorer (W×D×H: 30 cm × 30 cm × 50 cm) that allowed the retrieval of soft sediment with minimal disturbance. On deck, the sediment in the boxcore was randomly subsampled with PVC core liners (length:30 cm, inner diameter: 6.0 cm) where microprofiles were carried out in each replicate core (n=3-5) in a completely randomized fashion. At least two sediment cores were profiled at each of the four stations.<br><br>Sediment samples were flash frozen in liquid nitrogen during sampling. RNA was extracted by Elias Broman in the laboratory. Elias Broman also conducted bioinformatics and data analyses of the molecular data.
Timing and spatial scale	Samples were collected during 2018 April 23-26.
Data exclusions	No data was excluded from the analysis.
Reproducibility	The data collected was not from laboratory experiments, but collected in situ.
Randomization	Samples were taken from stations monitored over several years in other published geochemical studies.
Blinding	We did not conduct a clinical study and bindings was therefore not relevant.

Did the study involve field work? ☒ Yes ☐ No

Field work, collection and transport

Field conditions	Bottom water temperature was ranging between 3.8 to 7.4 celsius.
Location	Eastern Gotland Basin from for stations<br>Coordinates and additional details are available in the Methods section.
Access and import/export	Sediment and water sampling with methods described above (data collection).
Disturbance	Sediment was sampled via a boxcorer that allowed the retrieval of soft sediment with minimal disturbance.

Reporting for specific materials, systems and methods

We require information from authors about some types of materials, experimental systems and methods used in many studies. Here, indicate whether each material, system or method listed is relevant to your study. If you are not sure if a list item applies to your research, read the appropriate section before selecting a response.

Materials & experimental systems

n/a	Involved in the study
☒	☐ Antibodies
☒	☐ Eukaryotic cell lines
☒	☐ Palaeontology
☒	☐ Animals and other organisms
☒	☐ Human research participants
☒	☐ Clinical data

Methods

n/a	Involved in the study
☒	☐ ChIP-seq
☒	☐ Flow cytometry
☒	☐ MRI-based neuroimaging
